# Temperature-Modulated Changes in Thin Gel Layer Thickness Triggered by Electrochemical Stimuli

**DOI:** 10.1021/acs.langmuir.2c03228

**Published:** 2023-02-01

**Authors:** Klaudia Kaniewska, Kamil Marcisz, Marcin Karbarz

**Affiliations:** Faculty of Chemistry, Biological and Chemical Research Center, University of Warsaw, 101 Żwirki i Wigury Avenue, 02-089Warsaw, Poland

## Abstract

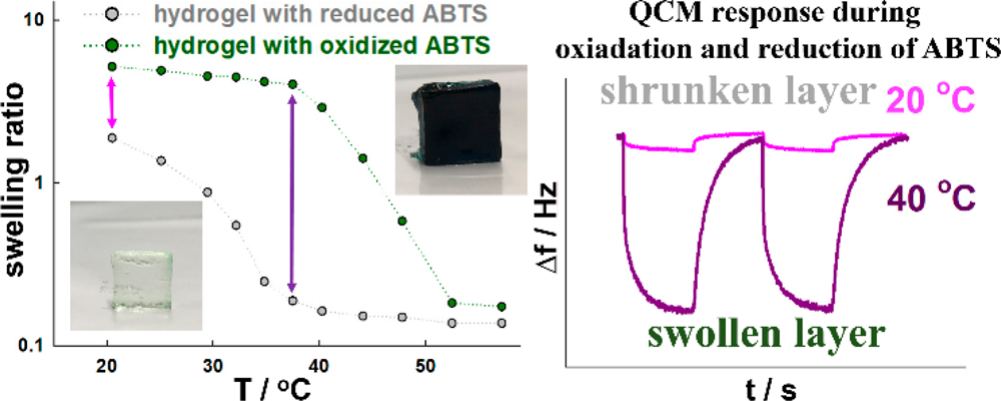

A series of thermoresponsive hydrogels containing positively charged groups in the polymeric network were synthesized and modified with the electroactive compound 2,2′-azino-bis(3-ethylbenzothiazoline-6-sulfonic acid) diammonium salt (ABTS). ABTS, which forms a dianion in aqueous solutions, acts as an additional physical cross-linker and strongly affects the swelling ratio of the gels. The influence of the amount of positively charged groups and ABTS oxidation state on the volume phase transition temperature was investigated. A hydrogel that possesses a relatively wide and well-defined temperature window (the temperature range where changes in the ABTS oxidation state affects the swelling ratio significantly) was found. The influence of the presence and oxidation state of ABTS on mechanical properties was investigated using a tensile machine and a rheometer. Then, a very thin layer of the gel was deposited on an Au electrochemical quartz crystal microbalance with dissipation (EQCM-D) electrode using the electrochemically induced free radical polymerization method. Next, chronoamperometry combined with quartz crystal microbalance measurements, obtained with an Au EQCM-D electrode modified by the gel, showed that the size of the thin layer could be controlled by an electrochemical trigger. Furthermore, it was found that the electrosensitivity could be modulated by the temperature. Such properties are desired from the point of view construction of electrochemical actuators.

## 1 Introduction

Polymeric gels are soft materials that consist of a hydrophilic network, formed by various natural and/or synthetic polymers cross-linked by covalent and/or non-covalent interactions and filled with an aqueous solvent. A high solvent content and solid consistency make hydrogels combine the properties of solids and liquids. The polymer network “immobilizes” the liquid phase, causing it to lose its fluidity. As a result, at the macroscale, the three-dimensional gel network retains its shape, stores mechanical energy, and can be subjected to deformation interactions. However, at the microscale, small molecule/ion transport processes occur in the liquid phase of the gel and may be only slightly impeded by the polymer network. As a result of their unique properties, these materials are very interesting and well in line with current trends in materials research. This is evidenced by the growing number of publications describing the properties of such gels and the possibilities of their application.^1−4^ In addition, as a result of their sensitivity to external stimuli, polymer gels are classified as “smart” materials.^5−7^ In response to external stimuli, they undergo a volume phase transition (VPT). The VPT process involves the alteration of the gel from the swollen phase to the shrunken phase or reverse.^8−10^ The reversible change in the volume of the gel during the phase transition can be up to several orders of magnitude, resulting in significant changes in its properties. The varied triggers can induce the volume changes of the hydrogels. Thus far, the most thoroughly investigated were hydrogel materials sensitive to the temperature and pH.^11−14^ Currently, attention is being paid to hydrogels sensitive to electric triggers. These electrosensitive hydrogels^15−19^ are very interesting materials for the construction of, for example, an advanced system of drug delivery, artificial muscles, actuators, micropumps, microvalves, and strain sensors.^20−27^

One group of electroresponsive gels that is of particular interest in the context of this work is the group of hydrogel materials consisting of electroactive units. These units may undergo redox reactions and, thus, affect the swelling ratio of the gel.

Two ways of obtaining electroactive hydrogels can be indicated: either the electroactive sites can be covalently bound to polymer chains, or the strong physical interactions between electroactive species and the network can be involved. In the case of chemically linked redox groups, the hydrophilicity of the polymer network can be altered by the changing of the oxidation state of electroactive groups. It can lead to significant changes in a swelling ratio (volume of a gel),^28−31^ whereas in the case of physically bounded redox groups, the change in the oxidation state of electroactive groups additionally affects the interaction between this groups and the polymer network. Therefore, the created physical cross-linking point can be strengthened or broken, resulting in volume changes.^32,33^ Hydrogel systems where the redox reaction can produce mechanical energy, by altering the volume/thickness or banding the hydrogel, are particularly interesting from the point of view of construction actuators, artificial muscles, or soft robotics.^22,34−38^

In addition, anchoring gel layers on conducting surfaces makes it possible to trigger volume changes by imposing an appropriate potential.^39−41^ For example, Takada et al. used the electrostatic interaction between poly(acrylic acid) (pAA) gel and Cu^2+^ to obtain an actuator.^42^ The complexation of positively charged and redox-acive Cu^2+^ ions by carboxylic groups from polymer chains takes place and leads to the gel layer shrinkage. Then, the electrochemical reduction of Cu^2+^ to Cu^0^ led to an increase in the swelling ratio of the gel layer. Kaniewska et al. also used copper(II) ions as an additional physical cross-linker in a polyacrylate hydrogel.^43^ A very thin negatively charged hydrogel layer was deposited on the quartz crystal microbalance (QCM) electrode surface through the electrochemically induced free radical polymerization method. Then, Cu^2+^ cations were accumulated in the very thin layer. The presence of copper ions led to shrinkage of the layer as a result of the formation of additional cross-linking points. However, most interestingly, the reduction process of Cu(II) resulted in the formation of Cu(I) rather than Cu^o^, as had been presented by Takada et al.^42^ As a consequence, the Cu(I) ↔ Cu^o^ process caused the reversible shrinking/swelling transformation of the hydrogel layer. Marcisz et al. showed an electrosensitive layer based on negatively charged polymer poly(sodium acrylate).^44^ The positively charged hexaammineruthenium(II)/(III) was added to solution and served as the additional cross-linkers. Tatsuma et al. synthesized a gel based on *N*-isopropylacryamide and vinylferrocene (VFc).^45^ The thermosensitive gel was attached to the electrode by pre-modifying the surface with compounds containing vinyl groups. It was found that the VPT temperature strongly depends upon the oxidation state of ferrocene groups, because for oxidized electroactive groups, the VPT temperature is much higher than in the reduced state. Then, at 30 °C, by applying an appropriate potential to the electrode, the transition from the swollen state to the shrunken state or vice versa was achieved.

Recently, we presented a positively charged thin gel layer soaked with an electroactive moiety with a negative charge. In the first step, the gold QCM electrode surface was modified with the thin gel layer and then redox-active 2,2′-azino-bis(3-ethylbenzothiazoline-6-sulfonic acid) diammonium salt (ABTS) was introduced. ABTS played the role of an additional cross-linking agent as a divalent anion. The electrochemical oxidation of ABTS led to the swelling process of the gel layer. Furthermore, the reduction of the oxidized ABTS moieties in the network caused a shrinking process. The obtained results were repeatable and reproducible and occurred suitably rapidly.^46^ The main aim of this paper was to obtain a new gel material in which the electrosensitivity can be modulated by the temperature. A series of gels, of which the dimensions can be altered by the redox reaction, was obtained. To this end, the same components as in our previous paper were used.^46^ The base monomer of the covalently cross-linked polymer network of the gels was *N*-isopropylacrylamide, which provided thermosensitivity. The second component was *N*-(3-aminopropyl)methacrylamide hydrochloride, which introduced charge. Then, ABTS was used as an electroactive additional physical cross-linker. It was aimed at achieving, by optimization of the hydrogel layer composition, the possibility of modulating the electrosensitivity of the gel by the temperature. Another important goal was to demonstrate the possibility of significantly changing the thickness of a gel layer anchored to a conductive substrate by applying an electric potential, as a very important property for the design of electrochemical actuators.

## 2 Materials and Methods

### 2.1 Materials

*N*-(3-Aminopropyl)methacrylamide hydrochloride (APMA), *N*-isopropylacrylamide (NIPA), *N*,*N*′-methylenebis(acrylamide) (BIS), *N,N,N',N'*-tetramethylethylenediamine (TEMED), ammonium persulfate (APS), 2,2′-azino-bis(3-ethylbenzothiazoline-6-sulfonic acid) diammonium salt (ABTS), and ascorbic acid were purchased from Aldrich. Sodium nitrate (NaNO_3_) and sodium hydroxide were purchased from POCh. NIPA was purified using recrystallization from a mixture of toluene/hexane (30:70, v/v). All other chemicals were used as received. The solutions were prepared using high-purity water obtained from a Milli-Q Plus/Millipore purification system (water conductivity of 0.056 μS cm^–1^).

### 2.2 Swelling Ratio Measurements

The swelling ratio was measured for a sample synthesized in 500 μm diameter glass capillaries. The preparation of hydrogel pieces for measurement included washing, cutting, and immersing of rod-shaped samples in water-coated cells. The parameter that was measured was the diameter; for this purpose, the optical microscope Zeiss Primo Vert supplied with a digital camera was used. The temperature was adjusted in the range of 20–60 °C using a circulating bath (PolyScience). The following equation has been applied *V*/*V*_o_ = (*d*/*d*_o_)^3^ to define the swelling ratio of hydrogel samples in varied conditions (*V* and *V*_o_ represent the equilibrium volume of the hydrogel and the initial gel volume; *d* denotes the diameter of the gel rod; and *d*_o_ is the diameter of the capillary in which the gel was synthesized). The precision of the gel rod diameter measurement was better than 3%.

### 2.3 Rheological Measurements

The Anton Paar MCR302 rheometer was used for dynamic shear rheology experiments using a set of 15 mm diameter parallel plates at a constant temperature of 20 °C. First, dynamic oscillatory strain sweep experiments were performed on the hydrogels to determine the limit of the linear viscoelastic region. The dynamic strain sweep (γ) was performed at a constant frequency, ω = 10 rad s^–1^, in the range from 0.01 to 1000%. Therefore, in all of the frequency sweep tests, the strain amplitude (γ) was fixed at 0.5% (within the linear viscoelastic range, which was small enough to avoid the nonlinear response and large enough to have a reasonable signal intensity), over a frequency range of 0.01–100 rad s^–1^. The temperature was controlled using a PolyScience circulating bath. To keep the desired constant temperature of hydrogel samples and minimize water evaporation during rheological measurements, a cap was used.

### 2.4 Mechanical Measurements

Compression tests were carried out using a Shimadzu EZ-SX universal tensile machine equipped with a 20 N load cell. Cylindrical hydrogel samples with a height of 5 mm were compressed at a compression rate of 5 mm/min at room temperature. Load and displacement data of the samples were collected in triplicate.

### 2.5 Electrochemical Measurements

A CH Instruments potentiostat (model CHI 400B) was used for electrochemical measurements. The three-electrode system was used, with a platinum wire as the counter electrode, a saturated silver chloride as the reference electrode, and quartz crystal microbalance with dissipation (QCM-D) Au as the working electrode. A modified electrochemical cell by the manufacturer was employed.

### 2.6 QCM-D Measurements

QCM-D measurements were performed with a QEM 401 (Q-Sense, Biolin Scientific) instrument equipped with 4.95 MHz AT-cut gold-coated quartz crystals. The QCM-D Au electrode was cleaned with a hot Piranha solution to remove organic pollutants, rinsed with water, and then dried with ethanol. Next, the electrode was mounted in the electrochemical cell. Data from the QCM-D measurment were used to calculate the changes in the thickness of the layers attached to the surface of the QCM electrode using the software, Dfind, by the manufacturer with the included viscoelastic Voigt-based model.

### 2.7 Scanning Electron Microscopy (SEM) Measurements

The morphology of the gel samples was analyzed using a Zeiss Merlin field emission scanning electron microscope. Before taking the micrographs, the macrogel samples were lyophilized using Labconco FreeZone lyophilizer at −82 °C and under a pressure of 0.03 mbar. The samples, prior to analysis, were coated with a thin ca. 3 nm layer of Au–Pd alloy using a mini-sputter coater from Polaron SC7620.

### 2.8 Synthesis of p(NIPA–*X*%APMA) Bulk Gel

The polymerization of p(NIPA–*X*%APMA) gels was made by the free radical method. The total concentration of monomers was 1000 mM, where the concentration of BIS was constant and equal to 1 mol % and APMA and NIPA varied at 1, 2.5, 5, and 10 mol % and 98, 96.5, 94, and 89 mol %, respectively. First, the pre-gel solution was deoxygenated, and then initiator APS (1.88 mM) and accelerator TEMED (32 mM) were added to start the polymerization reaction, which was carrying out for 1 day at 5 °C. The pH of the pre-gel solution was ca. 3. After 24 h, synthesized hydrogels were removed from glass and placed in deionized water for 1 week to remove any unreacted substrate. During this time, water was exchanged daily. The hydrogels were synthesized as rods with 500 μm diameter for optical microscope measurement and as rods with 25 mm diameter for mechanical investigations.

The gel samples for rheological measurements were first soaked with ABTS_red_ and then oxidized by Na_2_S_2_O_8_. All samples (p(NIPA–5%APMA), p(NIPA–5%APMA)–ABTS_red_, and p(NIPA–5%APMA)–ABTS_ox_) were washed with water and then cut into slices with 1.5 mm height and 15 mm in diameter.

### 2.9 Synthesis of the p(NIPA–5%APMA) Gel Layer on the QCM-D Electrode Surface

The modification of the Au QCM-D electrode surface with a p(NIPA–5%APMA) layer was performed via electrochemically induced free radical polymerization. The monomer concentration was equal for a base monomer NIPA at 658 mM, 35 mM for APMA, and 7 mM for a BIS cross-linking agent. Because the electrochemical reduction process was involved in generating the radicals, only the addition of the initiator was required (20 mM of APS). To provide conditions for diffusion transport, the monomers were dissolved in 0.2 M NaNO_3_ and alkalized with NaOH to ca. pH 9 to partially deprotonate the amine groups of APMA. The cell temperature was set at 20 °C. The electrosynthesis was usually terminated after 15 voltammetric cycles when the shift/drop of frequency of the third overtone had reached ca. 800 Hz. The modified electrode was kept in deionized water if not used.

## 3 Results and Discussion

**Figure 1:**
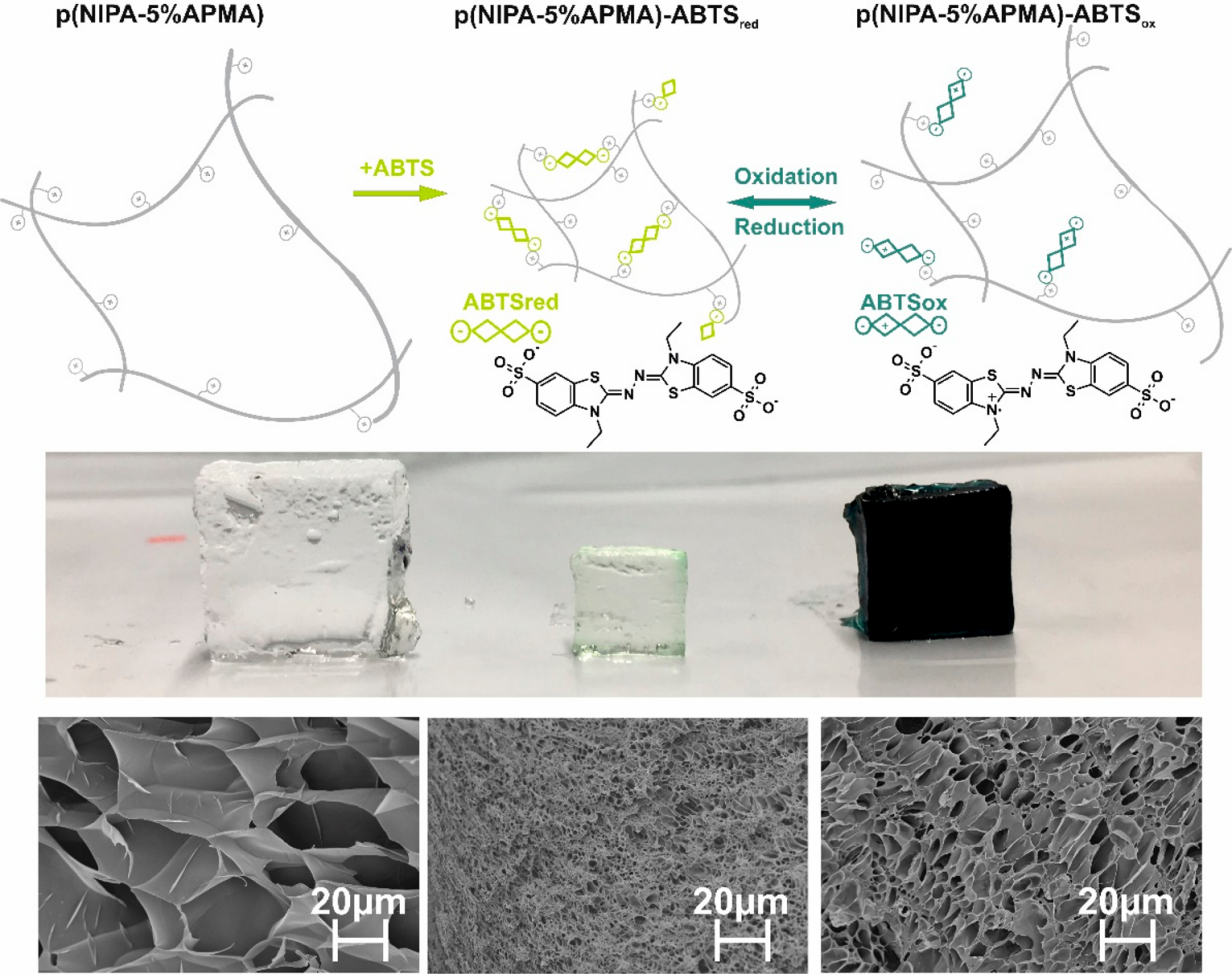
Schematic illustration of the p(NIPA–5%APMA) gel before and after ABTS modification and effect of ABTS oxidation state changes on the hydrogel structure as well as digital photography and scanning electron microscope images of hydrogel p(NIPA–5%APMA) before and after modification with ABTS and after oxidation.

A series of p(NIPA–APMA) hydrogels with positively charged polymer networks based on *N*-isopropylacrylamide (NIPA) and *N*-(3-aminopropyl)methacrylamide hydrochloride (APMA) cross-linked with *N*,*N*′-methylenebis(acrylamide) (BIS) was synthesized. Then, the gels were modified with 2,2′-azino-bis(3-ethylbenzothiazoline-6-sulfonic acid) diammonium salt (ABTS). ABTS has been chosen because it is electroactive and possesses in aqueous solutions a double charge related to deprotonated sulfonic groups (ABTS^2–^). Therefore, it can create physical cross-linking points (present in a gel beside chemical cross-links of BIS) by interaction with the positively charged polymer network. The scheme and photos of modification of p(NIPA–APMA) gels with ABTS^2–^ are presented in Figure 1. As seen, this process strongly affects the swelling ratio of the hydrogels. This suggests that additional cross-link points appeared. As mentioned, the thiazoline group of a divalent anion (ABTS^2–^) can be electrochemically or chemically oxidized (the five-member ring nitrogen atom is oxidized to the cation radical), which results in a decrease of a total charge of this species (ABTS^•^ ^–^).^47^ For the oxidation of p(NIPA–APMA)–ABTS, ammonium persulfate was used. After this process, the interaction of ABTS with the polymeric network weakened, which led to the disappearance of physical cross-links. This phenomenon has a significant influence on the swelling ratio and color of the p(NIPA–APMA)–ABTS hydrogel (the gel took on the characteristic color of the oxidized form of ABTS; see Figure 1). Also, it was found that this process is reversible, and after reduction of the hydrogel with ascorbic acid, the gel regained its original appearance.

**Figure 2:**
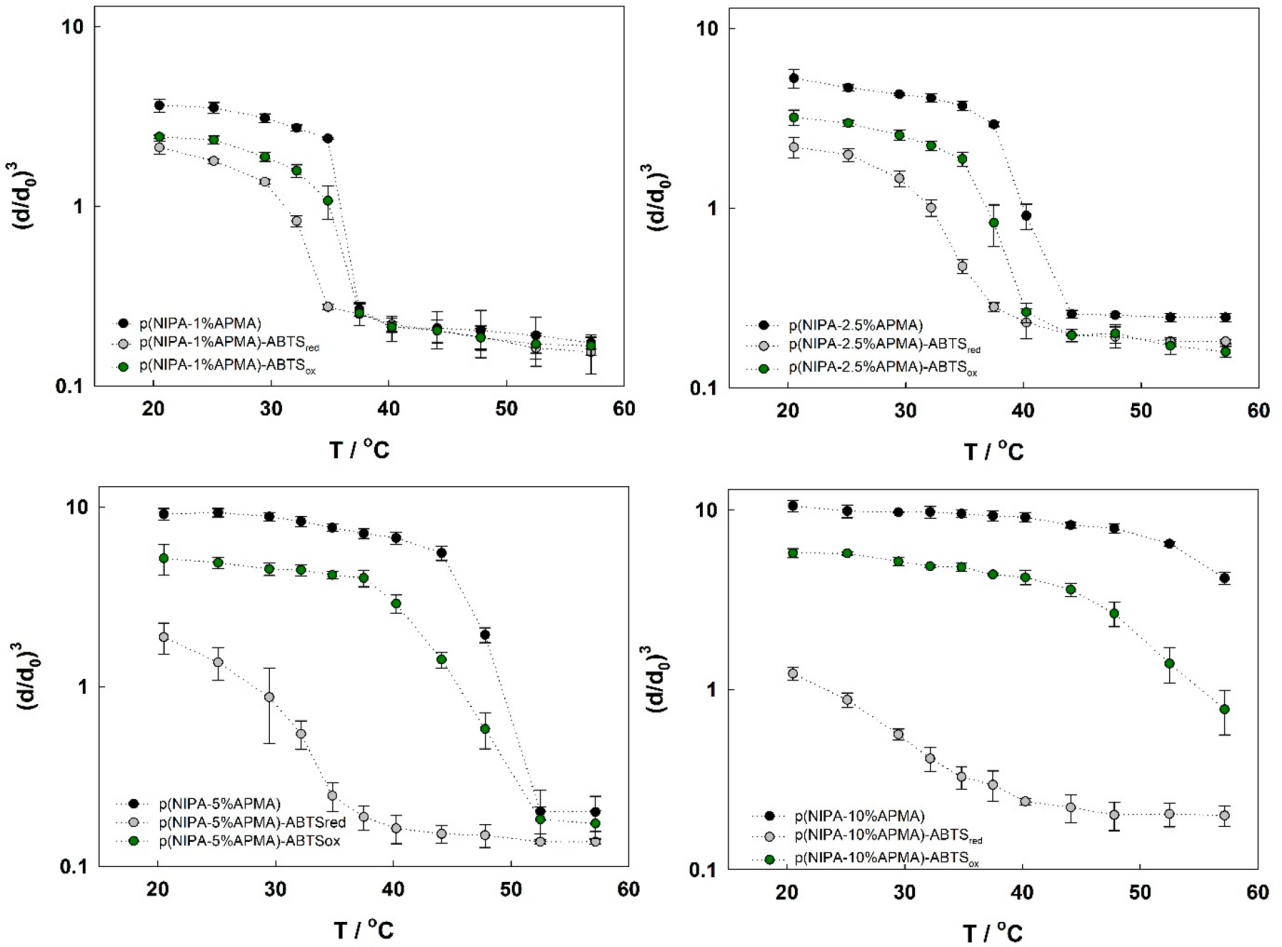
Swelling ratio changes dependent upon the temperature for hydrogels with various APMA contents, unmodified and modified with ABTS, and in reduced and oxidized states in water.

Then, the series of hydrogels with various amounts of amine groups (APMA monomer) was synthesized in capillaries. In this work, 1, 2.5, 5, and 10% APMA were selected, respectively. The influence of the presence of ABTS in its oxidation state and the temperature on the swelling behavior has been under investigation, and the results are presented in Figure 2. These experiments were carried out in solutions with ca. pH 6. Under these conditions, most of the amine groups of APMA were protonated, because the p*K*_a_ of APMA is greater than 10.^48^ As observed, the swelling ratio decreases in the order of p(NIPA–*X*%APMA) (black), p(NIPA–*X*%APMA)–ABTS_ox_ (green), and p(NIPA–*X*%APMA)–ABTS_red_ (gray) for all concentrations of amine groups. The introduction of ABTS results in a decrease in the swelling ratio and the temperature of the volume phase transition as a result of electrostatic attraction between the positively charged network and negatively charged ABTS. The oxidation of ABTS results in an increase in the swelling ratio and shifts the temperature of the volume phase transition to higher values. The observed differences in the swelling ratio and volume phase transition temperature become more pronounced with increasing APMA content. For the p(NIPA–5%APMA) gel, a relatively wide and well-defined temperature window (temperature range where the change of the oxidation state affects the swelling ratio significantly) was obtained. Therefore, for further study, the p(NIPA–5%APMA) gel as a double-sensitive material was selected.

**Figure 3:**
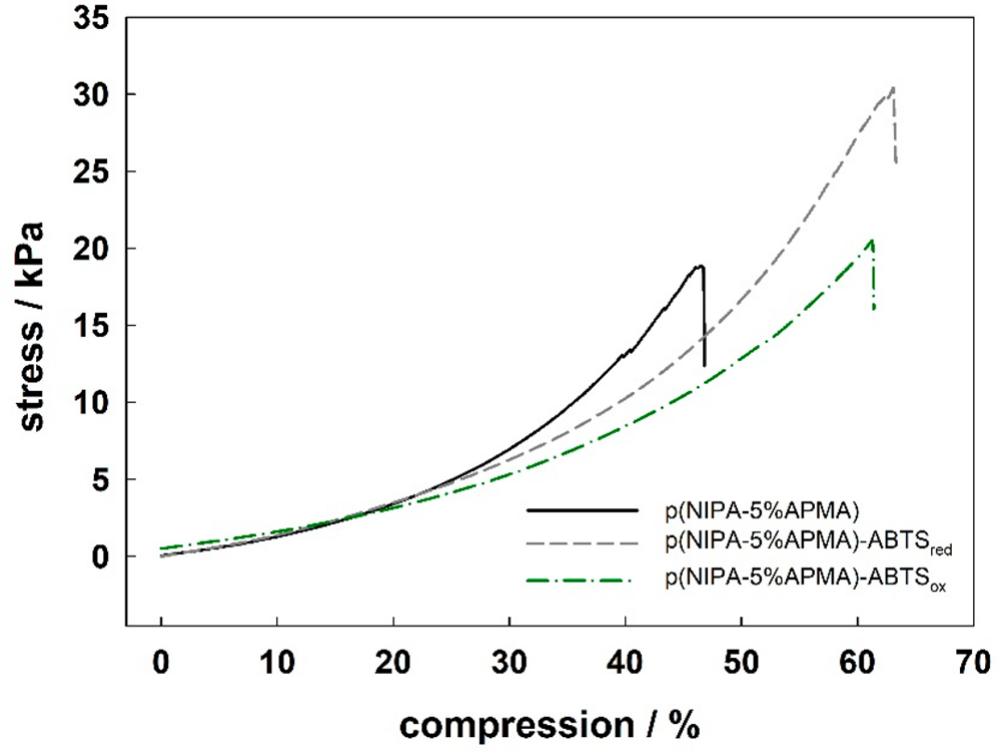
Compression strain–stress test for the p(NIPA–5%APMA) (black), p(NIPA–5%APMA)–ABTS_red_ (gray), p(NIPA–5%APMA)–ABTS_ox_ (green) hydrogels.

Next, the mechanical properties of the p(NIPA–5%APMA) gel were quantitatively evaluated by compressive tests (Figure 3). The value of compression stress at the break is the highest for p(NIPA–5%APMA)–ABTS_red_ and is 30.4 kPa; the value of compression stress at the break is the lowest for p(NIPA–5%APMA) and is 18.8 kPa; and the value of compression stress at the break is 20.5 kPa for p(NIPA–5%APMA)–ABTS_ox_. The compression at break was 46, 63, and 60% for p(NIPA–5%APMA), p(NIPA–5%APMA)–ABTS_red_, and p(NIPA–5%APMA)–ABTS_ox_. The introduction of additional physical cross-links led to an increase of the mechanical strength of the hydrogel. The energy is dissipated more effectively when physical and chemical cross-links co-exist and crushing of the structure for p(NIPA–5%APMA)–ABTS_red_ occurs for higher deformations.

**Figure 4:**
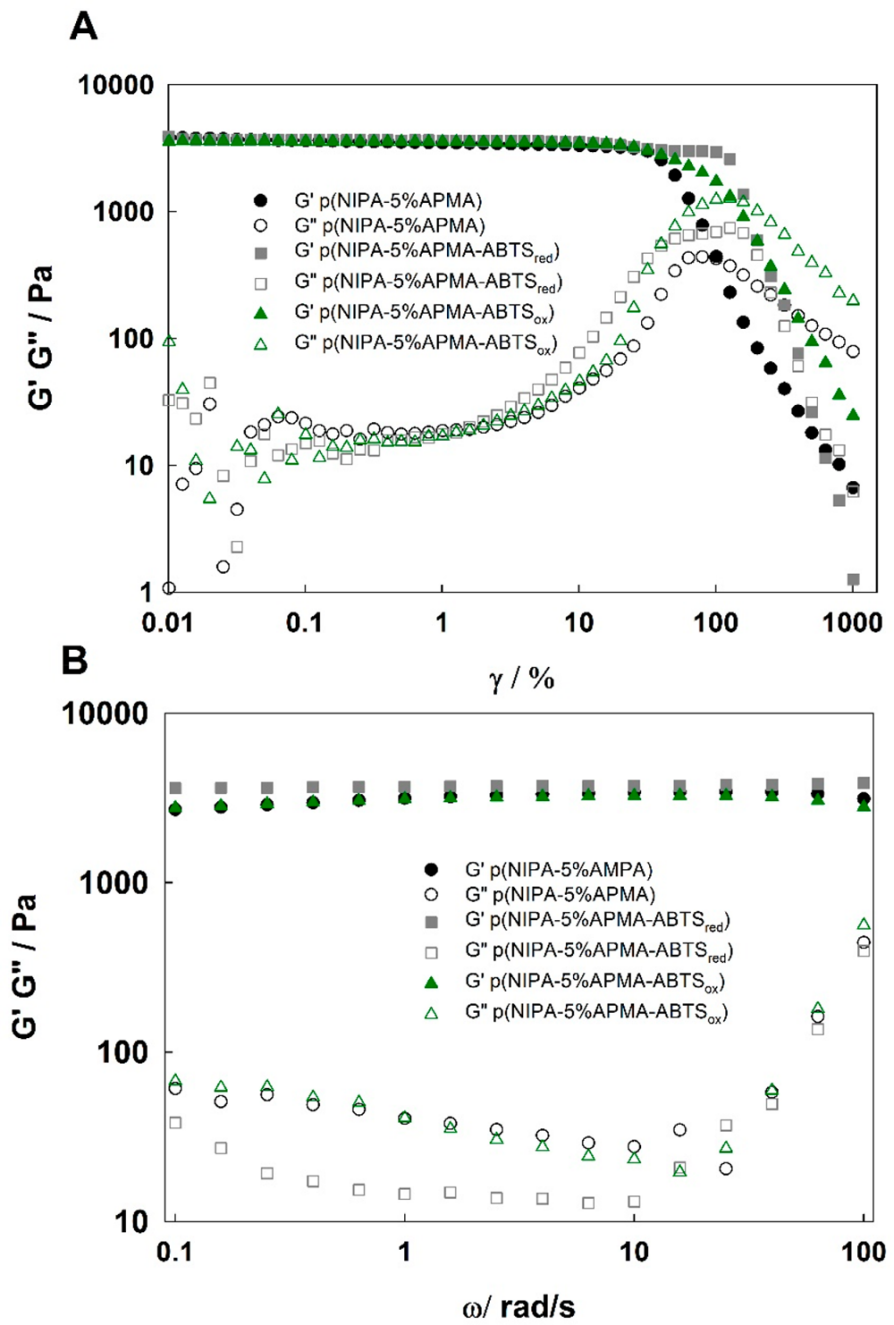
(A) Storage modulus *G*′ (solid symbols) and loss modulus *G*″ (hollow symbols) as a function of the shear strain and (B) storage modulus *G*′ (solid symbols) and loss modulus *G*″ (hollow symbols) as a function of the angular frequency for the p(NIPA–5%APMA) (black), p(NIPA–5%APMA)–ABTS_red_ (gray), and p(NIPA–5%APMA)–ABTS_ox_ (green) hydrogels.

Then, rheological measurements were performed to quantitatively determine mechanical properties of the hydrogels p(NIPA–5%APMA) before and after modification. Figure 4A shows the storage modulus (*G*′) and loss modulus (*G*″) as a function of the shear strain (γ) for a fixed frequency of 10 rad s^–1^. Relatively, a narrow linear viscoelastic region (LVR), where *G*′ and *G*′′ are independent of the shear strain, is seen for all of the gel samples investigated. Also, in this region, *G*′ is significantly higher than *G*″, which confirmed that the hydrogels were in a solid-like state. Further, when shear strain increases above the LVR region, the storage modulus decreased, while the loss modulus first increased and then decreased after the crossover point. This is the so-called type III behavior (weak strain overshoot^49,50^). The crossover point indicates the deformation at which the properties of gel material change from solid-like to viscous-fluid-like. In other words, it is the point at which material starts to flow or indicates internal structure crushing. In these cases, cracks in the polymer network appeared. For the p(NIPA–5%APMA)–ABTS_red_ gel, where two kinds of cross-links (chemical and physical) are present, the crossover points appear at a higher amplitude (250%) than for p(NIPA–5%APMA) and p(NIPA–5%APMA)–ABTS_ox_ (127 and 100%, respectively). This means that the double cross-linked polymer network of the p(NIPA–5%APMA)–ABTS_red_ gel is more mechanically robust.

Figure 4B shows the dynamic storage modulus and loss modulus at different angular frequencies. For this measurement, an amplitude sweep γ = 0.5% has been chosen from the linear viscoelastic region. For all obtained hydrogels, *G*′ is much larger than *G*″, indicating the solid-like and elastic nature of the hydrogels. As seen, the storage modulus is almost frequency-independent for the whole measured range, which is typical of covalently cross-linked gels, whereas the loss modulus changes. For the p(NIPA–5%APMA)–ABTS_red_ gel, *G*″ first decreases with increasing frequency, which suggests that energy can be dissipated efficiently in this region. This can be explained by the existence of dynamic bonds (cross-link points) between ABTS_red_ and the polymer network that can disassociate and reform, because for low frequencies, the time after strain application is long enough for a relaxation. For higher frequencies, the plateau region is observed. It is the region where the chemical bonds dominate and are responsible for the network behavior, because the stimulation time was too short to enable the network to effectively dissipate energy by dynamic bonds. In the case of the p(NIPA–5%APMA)–ABTS_ox_ and p(NIPA–5%APMA) gels, *G*″ first did not change much with increasing frequency, but after critical values for all gels, including p(NIPA–5%APMA)–ABTS_red_, a significant increase in *G*″ was found. For the highest frequencies, more viscous-like behavior is observed for all gels, with *G*″ approaching *G*′. This can be explained by the possibility of deformation of the polymer network.^51^ In addition, in all ω ranges, the storage modulus (*G*′) for p(NIPA–5%APMA)–ABTS_red_ is higher than that for p(NIPA–5%APMA) and p(NIPA–5%APMA)–ABTS_ox_ gels; e.g., for ω = 10 rad s^–1^, this value is 3790 Pa, and this value is 3400 and 3250 Pa for p(NIPA–5%APMA) and p(NIPA–5%APMA)–ABTS_ox_, respectively. This confirms that the p(NIPA–5%APMA)–ABTS_red_ gel has a higher mechanical stiffness. In addition, the loss moduli are very small, which means that the viscous part is negligible, as is typical for a hard gel.

**Figure 5:**
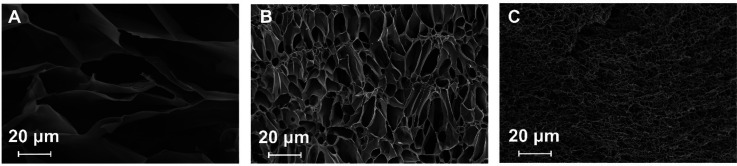
SEM images of lyophilized hydrogels (A) p(NIPA–5%APMA), (B) p(NIPA–5%APMA)–ABTS_ox_, and (C) p(NIPA–5%APMA)–ABTS_red_.

The changes in the gel structure upon modification with ABTS and its changing oxidation state were also confirmed by SEM photographs (Figure 5). To maintain the porous structure, the samples were freeze-dried. As clearly seen, the pore size is the greatest for unmodified p(NIPA–5%APMA) gels and the smallest for p(NIPA–5%APMA)–ABTS_red_, oxidation of ABTS leads to the increase in the pore size, but pores were smaller than for unmodified hydrogels. These observations are in good agreement with swelling behavior studies (Figure 2).

**Figure 6:**
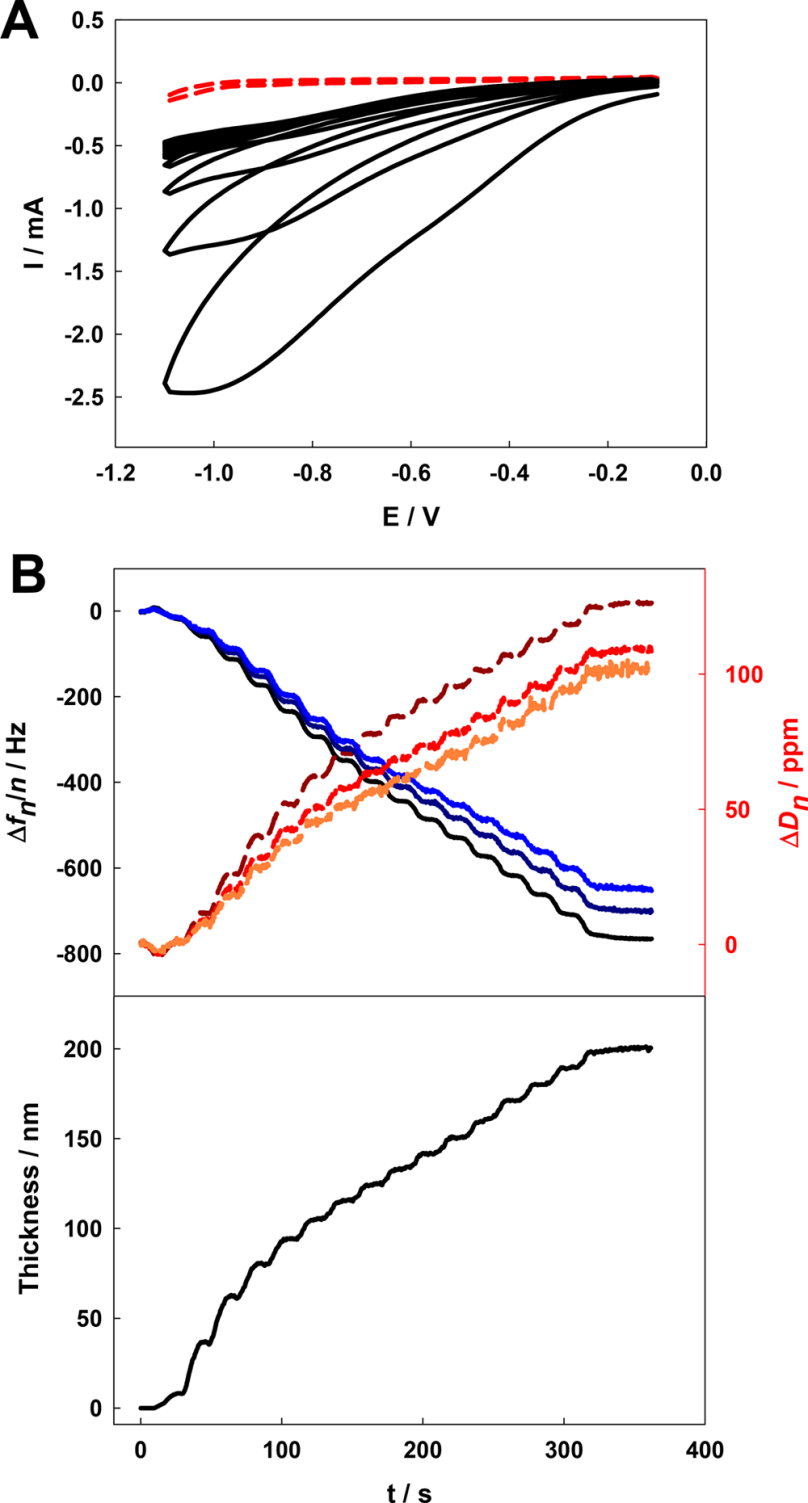
(A) Voltammograms obtained during deposition of the p(NIPA–5%APMA) layer on the QCM-D electrode surface. The solution contained NIPA, APMA, and BIS monomers with (black solid line) and without (red dashed line) an initiator. The pH of the solution was ca. 3. (B) Simultaneously registered frequency, Δ*f*, and dissipation, Δ*D*, changes during the electrode modification (curves for the third, fifth, and seventh overtones are presented). The black, navy blue, and blue solid lines, for frequency, and red, dark red, and orange dashed lines, for dissipation, represent the third, fifth, and seventh overtones, respectively. (Bottom) Calculated gel layer thickness growing during the modification process.

Then, to examine the possibility of triggering the volume change in the p(NIPA–5%APMA)–ABTS gel with an electric impulse, a thin layer of this gel was deposited on a QCM-D Au crystal electrode. To this end, the electrochemically induced free radical polymerization process was used. Typical voltammograms obtained during electrodeposition in the deoxygenated solution contain NIPA, APMA, BIS, and APS and are shown in Figure 6A. The relative concentrations of monomers were the same as those used in the synthesis of p(NIPA–5%APMA) macrogels. Scanning the potential in a range where reduction of APS occurs initiated the free radical polymerization reaction. The growth of the gel layer was monitored using the QCM-D technique. The polymerization was carried out until the third overtone reached a frequency of ca. 800 Hz, which was equivalent to ca. 15 voltammograms. The frequency and dissipation shifts are presented in Figure 6B.

These results allow for the thickness of the deposited viscoelastic hydrogel film on the electrode surface to be calculated. Layer thickness was calculated with a viscoelastic Voigt-based model (included in Dfind software) (Figure 6C). After electrodeposition, the electrodes were washed with water, and modified electrodes was kept in deionized water if not used. It should be stressed here that the calculated ca. 200 nm thickness is the thickness of the layer as prepared. After washing in water, the hydrogel layer thickness increased drastically up to 2000 nm.

**Figure 7:**
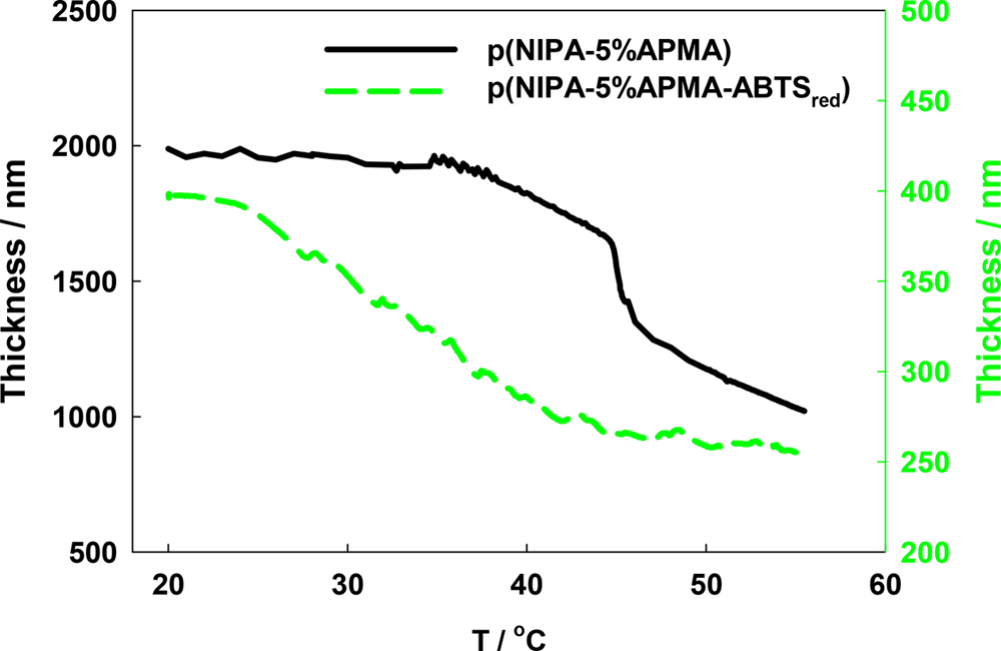
Temperature-dependent thickness of the p(NIPA–5%APMA) layer (black solid line) and p(NIPA–5%APMA)–ABTS_red_ layer calculated from the changes in frequency and dissipation using the Dfind software. The pH of the solutions was ca. 3.

Then, the thermoresponsive properties of the p(NIPA–5%APMA) gel layer on the QCM-D Au electrode surface with and without introduced ABTS were studied. The changes in layer thickness as a function of the temperature, calculated from frequency and dissipation shifts, are presented in Figure 7. The addition of a double-negatively charged redox probe ABTS to the positively charged polymer film leads to tremendous shrinkage of the layer from 2000 to 400 nm. Simultaneously, the shift of the volume phase transition to a lower temperature is observed. Results obtained for a thin layer are in agreement with the results obtained for the bulk gel (compare to Figure 2).

**Figure 8:**
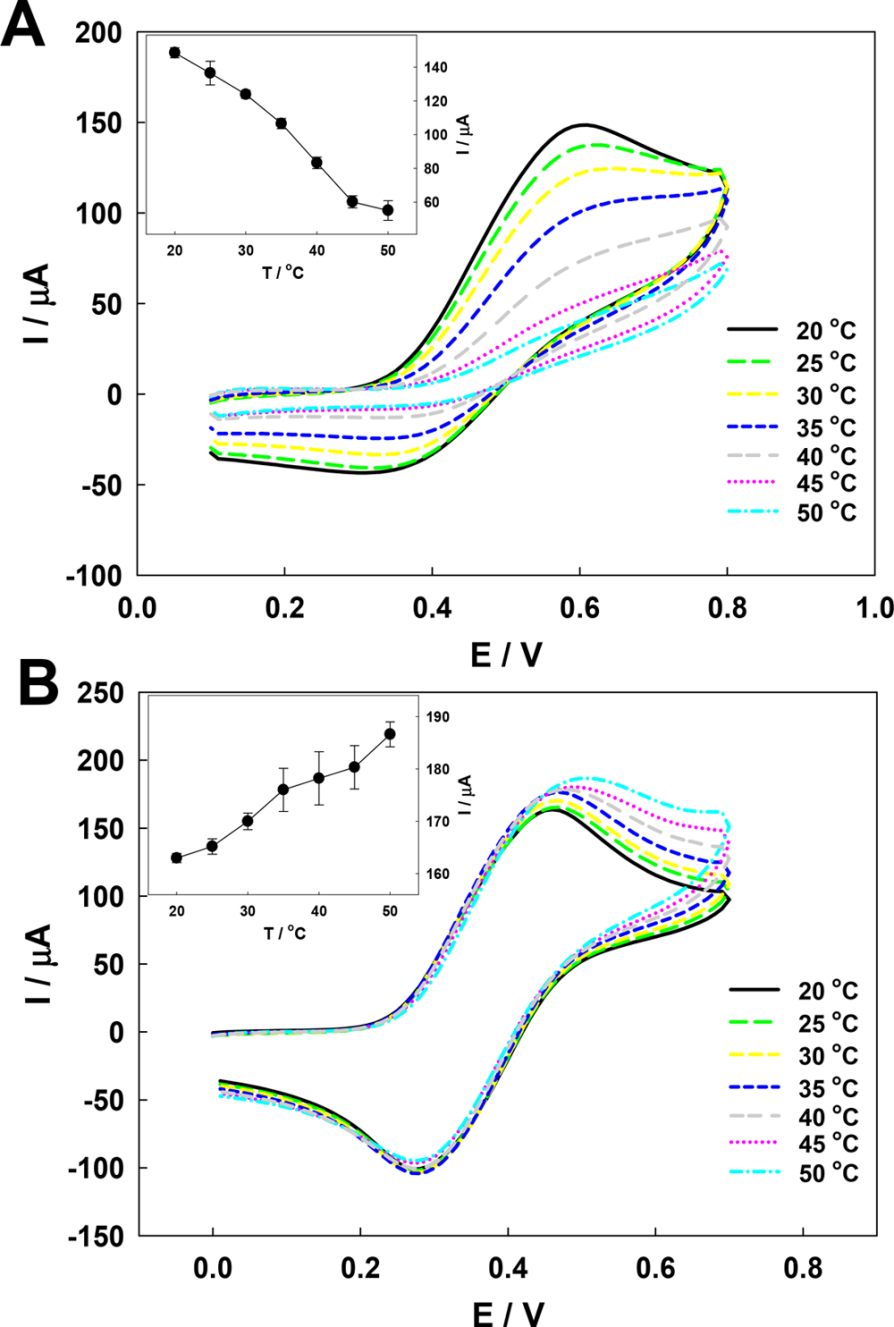
(A) Cyclic voltammograms obtained in 2 mM ABTS solution (20 mM NaNO_3_ supporting electrolyte) with the QCM-D electrode modified with the p(NIPA–5%APMA) gel layer at different temperatures. (Inset) Changes in the voltammetric peak current as a function of the temperature. (B) Cyclic voltammograms obtained in 2 mM ABTS solution (20 mM NaNO_3_ supporting electrolyte) with the QCM-D Au electrode at different temperatures. (Inset) Changes in the voltammetric peak current as a function of the temperature. *v* = 50 mV/s. The pH of the solutions was ca. 3.

Next, the electrochemical examination of the Au QCM-D electrode modified with the p(NIPA–5%APMA) layer was performed. For this purpose, the modified electrode was immersed in 2 mM ABTS solution containing 20 mM NaNO_3_ as the supporting electrolyte. The voltammograms were measured at various temperatures and are presented in Figure 8.

ABTS physically bound to the polymer network remains electroactive; in voltammograms, a pair of peaks characteristic of ABTS appeared. Both oxidation and reduction peak currents were dependent upon the temperature. An increase in the temperature caused a decrease in the peak current, which is related to deswelling of the polymer network and correlated well with the change in the gel layer thickness presented in Figure 7. Upon shrinkage, polymer chains become more rigid and the fraction of the polymer in the gel layer becomes higher. Both effects lead to difficulties in electron transport to the electrode surface and a drop in recorded currents. The shape of voltammograms requires a comment: the voltammograms are not well-shaped, and the oxidation current was substantially higher than the reduction current. This could be caused by the expelling of partially positively charged oxidized forms of ABTS from the positively charged polymer network. For comparison, voltammograms were recorded using a bare Au QCM-D electrode under the same conditions. As seen in Figure 8B, the voltammograms have the shape characteristic of a quasi-reversible electrode process, and the typical increase in the peak current as a result of the temperature rise is evident.

**Figure 9:**
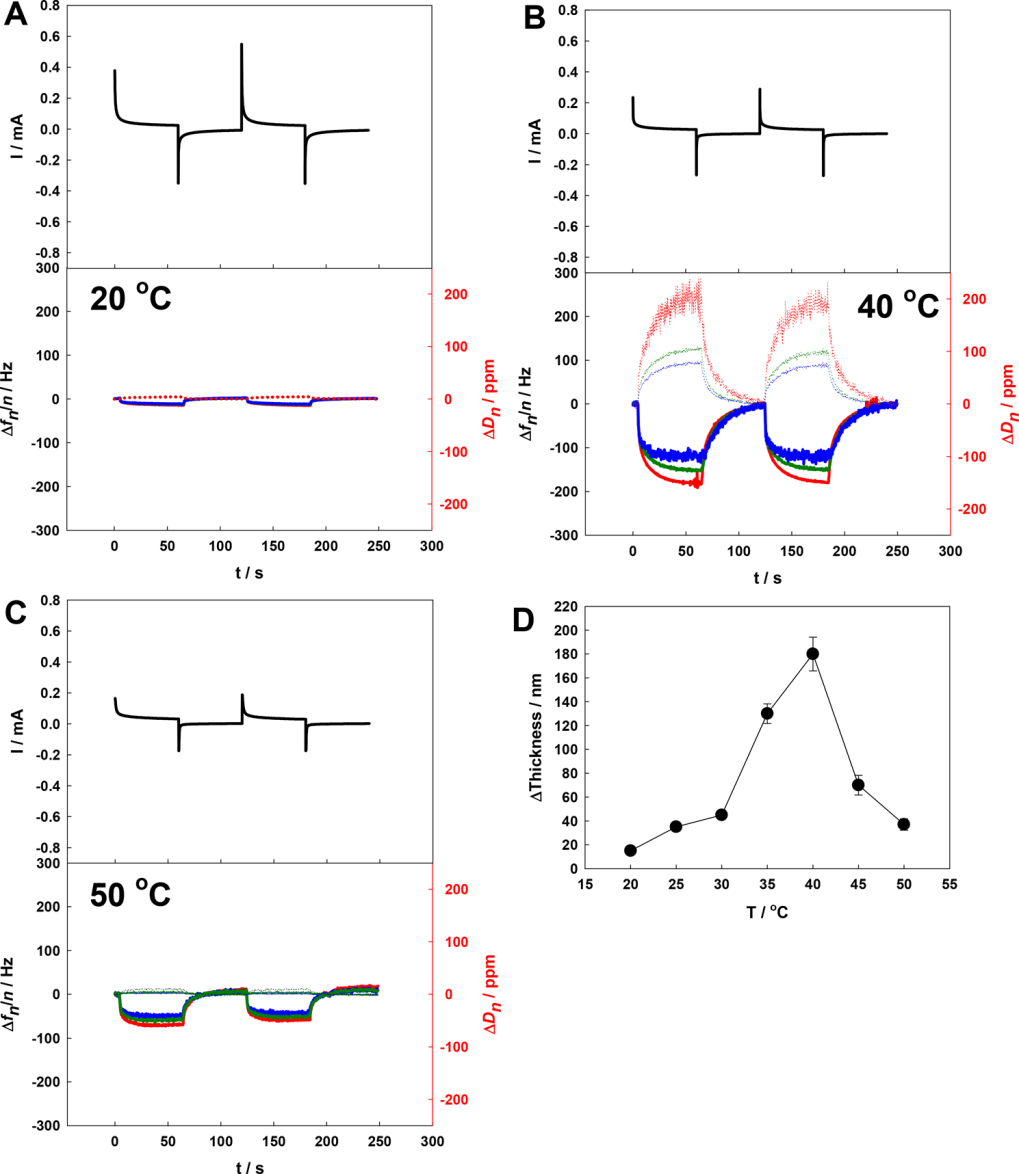
Multi-pulse chronoamperograms and simultaneously acquired frequency and dissipation changes obtained for the QCM-D electrode modified with the p(NIPA–5%APMA) layer obtained at different temperatures (A, 20 °C; B, 40 °C; and C, 50 °C) in 2 mM ABTS solution (20 mM NaNO_3_ supporting electrolyte). *E*_ox_ = 0.8 V, and *E*_red_ = 0.2 V. The red, green, and blue solid and dotted lines show frequency and dissipation energy changes for third, fifth, and seventh overtones, respectively. The pH of the solution was ca. 3. (D) Temperature-dependent p(NIPA–5%APMA) layer thickness changes, as a response to electrochemical oxidation of ABTS, calculated from frequency/dissipation shifts.

According to the data presented in Figure 2, there is a specific temperature range at which the volume of the p(NIPA–5%APMA) gel modified with ABTS depends upon the oxidation state of the electroactive moieties bound to the polymer network. Therefore, in the next step, the Au QCM-D electrode surface covered with a p(NIPA–5%APMA) gel layer with ABTS was examined with chronoamperometry and the QCM-D technique. The chronoamperograms with simultaneously registered quartz crystal frequency and dissipation shifts were obtained at various temperatures and are presented in Figure 9. As seen, the QCM-D response to the chronoamperometric oxidation/reduction of ABTS is strongly related to the temperature. At 20 °C (Figure 9A), only a small change in the frequency and dissipation shifts was observed. This is as expected, because at that temperature, reduced and oxidized forms are in the swollen state. At 40 °C (Figure 9B), oxidation of ABTS (divalent anion) led to a significant decrease in the frequency shift and increase in the dissipation. These changes show that the gel layer significantly changed the volume. Electrochemical reduction of the electroactive probe caused the opposite effect: quartz crystal frequency increases and dissipation decreases as a response to gel layer shrinkage. At this temperature, the p(NIPA–5%APMA) gel could exist in either the shrunken state or the swollen state depending upon the oxidation. In the oxidized form, the gel should be in the swollen state, and in the reduced form, the gel should be in the shrunken state. However, at 50 °C (Figure 9C), the QCM-D responses were visible, but changes were much smaller than at 40 °C because the gel layer in both oxidation states was in the shrunken state. Moreover, the QCM-D responses of the p(NIPA–5%APMA)–ABTS gel layer were reasonably fast, reversible, and repeatable. In Figure 9D, the thickness changes, calculated from QCM-D data, between reduced and oxidized states as a function of the temperature are presented. This clearly shows that the electrosensitivity of the gel layer strongly depends upon the temperature and that the temperature range where the change in the oxidation state affects the swelling ratio significantly (temperature window) is from ca. 35 to 40 °C.

An important issue is whether the gel layer is homogeneously/entirely oxidized or reduced during the chronoamperometry step. To determine this, the apparent diffusion coefficients for ABTS were first estimated using the Randles–Sevcik equation from the voltammograms shown in Figure 8A. The apparent diffusion coefficient values were found to be 1.6 × 10^–6^, 5.4 × 10^–7^, and 2.4 × 10^–7^ cm^2^/s at 20, 40, and 50 °C, respectively. Diffusion layer thicknesses were then estimated from the equation . It was found that, after 1, 30, and 60 s, the diffusion layer thicknesses were 12.6, 7.3, and 4.9 μm at 20 °C, 69.2, 40.1, and 27.0 μm at 40 °C, and 97.9, 56.74, and 38.1 μm at 50 °C, respectively. As seen, even after 1 s, the thicknesses of the diffusion layers are significantly bigger than the thickness of the gel layer. Thus, it can be concluded that, after the potential step, the gel layer is homogeneously oxidized or reduced. Therefore, the kinetics of frequency change is rather related to the kinetics of swelling/shrinking of the gel layer.

## 4 Conclusion

A series of double cross-linked hydrogels based on *N*-isopropylacrylamide and *N*-(3-aminopropyl)methacrylamide was successfully obtained. The positively charged network of the gels was chemically cross-linked with *N*,*N*′-methylenebis(acrylamide) and physically with dianion ABTS. It was found that the p(NIPA–5%APMA)–ABTS gel can exist in the swollen or shrunken state depending upon the oxidation state of ABTS across a relatively broad temperature range. Investigation of mechanical properties, in turn, showed that, when ABTS exists as a dianion (reduced form), the p(NIPA–5%APMA)–ABTS_red_ gel is harder and more mechanically durable. It was concluded that ABTS_red_ acts as an additional physical cross-linker and strongly affects the swelling ratio and mechanical properties of the gels. Finally, to demonstrate that the thickness of the hydrogel layer could be modulated using electrochemical techniques, the gold electrochemical quartz crystal microbalance with dissipation (EQCM-D) electrode was modified with a thin gel film using electrochemically controlled free radical polymerization. Then, using chronoamperometry combined with quartz crystal microbalance measurements, it was proven that the thickness of the hydrogel film could be controlled using an electrochemical trigger. In addition, the electrosensitivity of the p(NIPA–5%APMA)–ABTS gel layer strongly depends upon the temperature. In the range of the temperature from ca. 35 to 40 °C, electrochemically stimulated changes in the thickness of the gel layer were the highest.

The unique properties of stimuli-sensitive hydrogels combined with conducting surfaces open up new possibilities for the construction of novel electrochemical devices. With further refinement, we expect that gel materials whose size can be controlled by electrochemical triggers with a temperature-modulated response will lead to progress in the construction of electrochemical actuators, such as artificial muscles or electrochemical valves, as well as advanced active substance delivery systems.
